# Supplementary material for: Causal Relationship between Aging and Anorexia Nervosa: A White-Matter-Microstructure-Mediated Mendelian Randomization Analysis
Source: Biomedicines. 2024 Aug 16;12(8):1874. doi: 10.3390/biomedicines12081874 (PMC11351342; doi:10.3390/biomedicines12081874)
Supplement: Supplementary file 1 [file biomedicines-12-01874-s001.zip › Supplementary Figure/Figure S8/Leaveoneout plot of MR analysis for TL to ubm-b-1811.pdf]

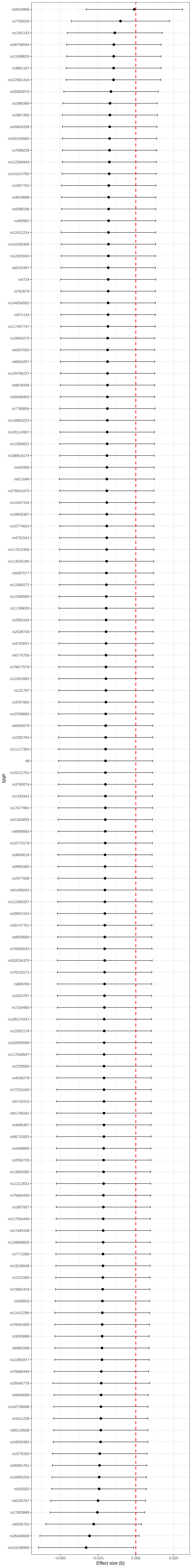
rs9419958
rs7705526
rs1291143
rs56799554
rs11699829
rs3891167
rs137901416
rs28363070
rs1985369
rs2967355
rs45604339
rs150150565
rs7099229
rs112394943
rs141214782
rs1907702
rs4616688
rs9398196
rs450962
rs12412214
rs142426306
rs12925933
rs8102497
rs4724
rs762679
rs144204502
rs871134
rs117407747
rs10840270
rs6007020
rs6054257
rs139795227
rs9878436
rs59409453
rs7790856
rs116863223
rs145114957
rs11584821
rs188918174
rs429358
rs611646
rs376641875
rs11557154
rs10845387
rs10774624
rs4731541
rs117512405
rs113525195
rs6587577
rs11085072
rs12369950
rs11769630
rs2555104
rs2538745
rs4743037
rs6776756
rs79977579
rs12451892
rs131797
rs3767952
rs10768683
rs6584579
rs2282764
rs11117354
All
rs10112752
rs3785074
rs1332941
rs17677991
rs41304832
rs6669563
rs10773176
rs9600019
rs9955360
rs2977608
rs61405042
rs111950327
rs28502153
rs55747751
rs6659669
rs76065543
rs202034370
rs76219171
rs869785
rs1023767
rs7164950
rs185174247
rs12932179
rs182059586
rs117630647
rs2230590
rs4530278
rs77231040
rs5742915
rs61748181
rs4695407
rs66731853
rs4498805
rs2056726
rs13062095
rs11212631
rs75664430
rs1957937
rs117034449
rs17445108
rs139669835
rs7772289
rs13230646
rs7221585
rs73581419
rs939916
rs11412296
rs78491606
rs3093888
rs6881568
rs11991877
rs76666449
rs35640778
rs9940099
rs142730696
rs1611236
rs80116508
rs34550383
rs2276182
rs56061761
rs10905255
rs932002
rs8105767
rs17803849
rs6536702
rs35446936
rs143190905
SNP
-0.050
-0.025
0.000
0.025
Effect size (b)
